# The MARS PETCARE BIOBANK protocol: establishing a longitudinal study of health and disease in dogs and cats

**DOI:** 10.1186/s12917-023-03691-4

**Published:** 2023-08-17

**Authors:** Janet E. Alexander, Serina Filler, Philip J. Bergman, Claire E. Bowring, Laura Carvell-Miller, Brenda Fulcher, Richard Haydock, Teresa Lightfoot, Darren W. Logan, Talon S. McKee, Tracy Mills, JoAnn Morrison, Phillip Watson, Colby Woodruff, The Mars Petcare Biobank Project Team, Graham Atkinson, Vincent Biourge, Konstantin Bobov, Aletha Carson, Alison Colyer, Kelly Cooper, Geert De Meyer, Rebecca Chodroff Foran, Tamara Gates, Kristi Grace, Lieve Goubert, Cassie Kresnye, Mary Kurian, Christian Leutenegger, Eric Lovvorn, Silvia Miret Catalan, Kay O’Donnell, Omar Ondoy, Rhiannon Reynolds, Katy Smith, Stacy Smith

**Affiliations:** 1 Waltham Petcare Science Institute, Waltham On the Wolds, Leicestershire, UK; 2 VCA Clinical Studies, 12401 West Olympic Blvd, Los Angeles, CA USA; 3 BluePearl Science, 2950 Busch Lake Blvd, Tampa, FL USA; 4 Banfield Pet Hospital, 18101 SE 6Th Way, Vancouver, WA USA; 5 Antech Diagnostics, 17620 Mount Herrmann St, Fountain Valley, CA USA

**Keywords:** Repository, Feline, Canine, Risk factors, Prospective, Cohort, Longitudinal, Disease, Genomic, Microbiome, Biomarker

## Abstract

**Background:**

The veterinary care of cats and dogs is increasingly embracing innovations first applied to human health, including an increased emphasis on preventative care and precision medicine. Large scale human population biobanks have advanced research in these areas; however, few have been established in veterinary medicine. The MARS PETCARE BIOBANK™ (MPB) is a prospective study that aims to build a longitudinal bank of biological samples, with paired medical and lifestyle data, from 20,000 initially healthy cats and dogs (10,000 / species), recruited through veterinary hospitals over a ten-year period. Here, we describe the MPB protocol and discuss its potential as a platform to increase understanding of why and how diseases develop and how to advance personalised veterinary healthcare.

**Methods:**

At regular intervals, extensive diet, health and lifestyle information, electronic medical records, clinicopathology and activity data are collected, genotypes, whole genome sequences and faecal metagenomes analysed, and blood, plasma, serum, and faecal samples stored for future research.

**Discussion:**

Proposed areas for research include the early detection and progression of age-related disease, risk factors for common conditions, the influence of the microbiome on health and disease and, through genome wide association studies, the identification of candidate loci for disease associated genetic variants. Genomic data will be open access and research proposals for access to data and samples will be considered. Over the coming years, the MPB will provide the longitudinal data and systematically collected biological samples required to generate important insights into companion animal health, identifying biomarkers of disease, supporting earlier identification of risk, and enabling individually tailored interventions to manage disease.

**Supplementary Information:**

The online version contains supplementary material available at 10.1186/s12917-023-03691-4.

## Background

Dogs and cats hold a unique position among domesticated animals, in sharing the home environment in which humans live. In the USA, it is estimated that 54% of households include a dog and 35% a cat [1], and in the United Kingdom this number is around 30% for both dog and cat ownership [2]. The veterinary care that cats and dogs receive has advanced in recent years in embracing many of the innovations applied to human health, including greater emphasis on preventative care and precision medicine [3, 4]. In human healthcare, advances in these areas have been strongly supported by large scale population biobanks [5]. The approach of collecting data and biological samples from individuals over their lifetimes has enabled scientific discoveries that have changed our understanding of why and how diseases develop [6]. For example, a model built to estimate the 2-year probability of lung cancer diagnosis using data from 502,321 UK biobank participants had excellent discriminatory power that was superior to standard lung cancer screening eligibility criteria [7]. The Baltimore Longitudinal Study of Aging [8] provides another example; this data aided research into effects of age-associated physiological changes on disease development, most notably the recognition of arterial stiffening as an important risk factor for cardiovascular disease [9].

Many other human biobanking and cohort studies have been active for several decades including the Framingham Heart Study, the Nurses' Health Study, the UK Biobank, and the Avon Longitudinal Study of Parents & Children [10–13]. These initiatives have had an important influence on the understanding of disease risk; however, few longitudinal studies have been established in companion animals. Some examples; such as the Golden Retriever Lifetime Study and the Dogslife study focus on particular breeds [14, 15]. In contrast, the NIH-funded Canine Longevity Consortium Dog Aging Project aims to identify genetic and environmental factors affecting longevity in the general canine population [16]. These studies have already contributed to veterinary knowledge by identifying risk factors for canine obesity, orthopaedic disease, cognitive dysfunction, and gastrointestinal illness [17–19]. In domestic cats, the lack of long-term health-related data is a limiting factor in veterinary research. One of the few long running feline cohort studies is the Bristol Cats Study [20]; a birth cohort of 2203 pet kittens living in the UK with prospective data collection from owners and veterinarians to explore links between common disorders and environmental exposures [21].

A lack of well annotated companion animal biological samples has also been highlighted as a limiting factor in veterinary research. To alleviate this, a number of veterinary biobanking initiatives are concentrating on specimen collection. Some are disease targeted, several of which have a focus on cancer for example; the Pfizer-canine comparative oncology and genomics consortium biospecimen repository [22], the long running Swiss Canine and Feline Cancer Registries [23] and the Australian Veterinary Cancer Biobank; a network of 50 veterinary clinics and pathology laboratories [24]. Other initiatives include the Canine Brain and Tissue Biobank at Etövös Lorand University, Budapest [25], focused on canine cognitive dysfunction and the Italian Canine and Feline biobank focused on inherited disease [26]. Other biobanks, housed within academic departments, often store opportunistically collected biological samples e.g., The VetBiobank, Vetmeduni Vienna [27]. Currently, the only International Society for Biological and Environmental Repositories (ISBER) accredited companion animal biobank is the Cornell University Veterinary Biobank; a biospecimen collection and archiving resource primarily focused on genetic disorders with collections often customized for individual studies e.g., The Dog Aging Project [28, 29].

While it has been suggested that evidence-based veterinary practice could benefit from biobanks to bridge the gap between patient care and clinical innovation, there are several obstacles to establishing general population biobanks [30]. These include; referral bias in pets attending specialist centres [31], difficulties accessing healthy animals attending primary care practices, challenges in transporting biological samples over wide geographical areas in a timely manner and the lack of standardised electronic medical record (EMR) coding across veterinary practices. The range of businesses that exist within Mars Petcare represent a unique opportunity to overcome many of these issues; its network of primary care and referral veterinary hospitals utilising a small number of EMR systems, veterinary diagnostics laboratories providing sample transport, handling and analysis, direct-to-consumer products that capture health and wellbeing information, and research centres to integrate and analyse the resulting data.

A protocol has been developed to recruit large numbers of initially healthy pets and follow them over time, providing opportunities for both cross-sectional and longitudinal research. The aim of the MPB is therefore to establish a unique longitudinal study and biobank, recruiting 20,000 healthy domestic cats and dogs, at a target rate of approximately 1000 a year, over an initial ten-year period. As some recruited pets will remain healthy, and others will go on to develop health conditions, findings from the study will enable the identification of ways to prevent and treat disease as well as delivering insights that may support healthy growth, development, and ageing. This approach will also provide a platform to move towards precision veterinary healthcare.

## Methods/design

### Study design

The MPB is a prospective study recruiting healthy dogs and cats attending Mars Veterinary Health (MVH) hospitals (VCA™ Animal Hospitals, Banfield® Pet Hospitals and BluePearl™ Specialty and Emergency Pet Hospitals) in the United States. The study launched recruitment of dogs in March 2022 and cats in June 2022 (Protocol version 4.1, June 28^th^, 2022). Informed consent is obtained from the pet owners, and an annual veterinary medical exam with routine blood biochemistry and haematology carried out. Blood and faecal samples are collected and banked annually. Owners also consent to completing regular quality of life (QoL) and diet, health, and lifestyle (DHL) surveys and allow the biobank access to their pet’s EMR. As an introduction to each survey a statement is made indicating that all information provided is treated confidentially and that by returning the survey the owner consents to the information being used by the MPB. No owner-specific data (gender, age, demographics, location etc.) are recorded other than the presence and number of children in the household, although the owner can opt out of providing this information if they wish. Location is not defined in greater resolution than the hospital attended.

### Setting

**Fig. 1:**
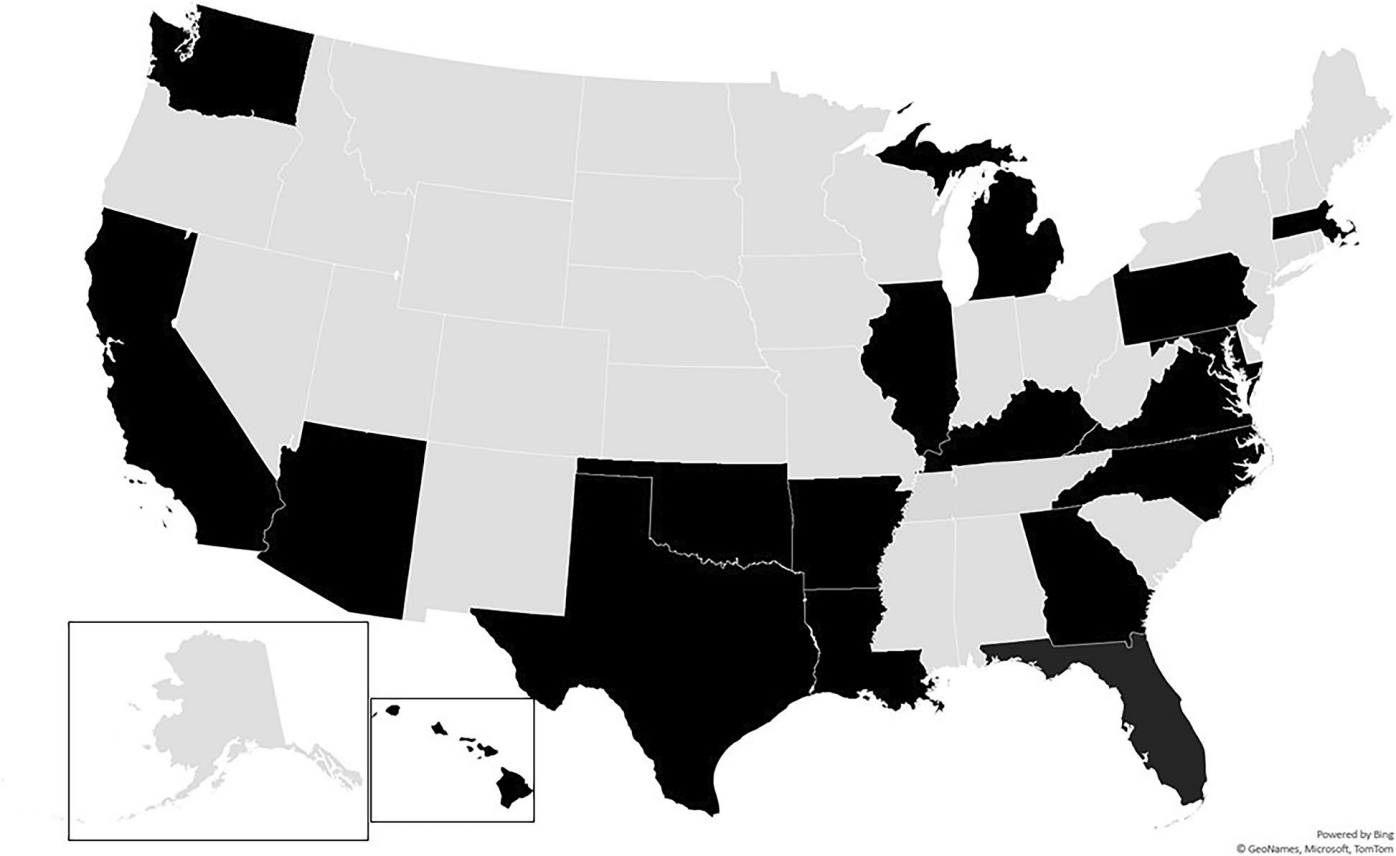
Graphical representation of states containing MARS PETCARE BIOBANK recruiting veterinary hospitals at launch. States with participating hospitals coloured in black and without participating hospitals in light grey. This map was generated in Microsoft Excel, powered by Bing, © GeoNames, Microsoft, Navinfo, TomTom, Wikipedia

At launch, recruitment of pets is through MVH primary care hospitals, with the addition of blood donors and staff pets attending BluePearl Specialty and Emergency Pet Hospitals. Initially, 51 hospitals enrolled covering 19 states: Arizona, Arkansas, California, Florida, Georgia, Hawaii, Illinois, Kentucky, Louisiana, Maryland, Massachusetts, Michigan, North Carolina, Oklahoma, Pennsylvania, Tennessee, Texas, Virginia, and Washington (Fig. 1). It is expected that further hospitals will be added to meet the recruitment targets and to achieve a geographically diverse population. Data recording, withdrawal, and adverse effects reporting are through the study’s electronic data capture system (EDC) (Castor EDC, Amsterdam, the Netherlands). Antech® Diagnostics (Fountain Valley, CA, USA) are responsible for the transport, processing, primary analyses, and storage of biological samples.

### Study participants

Owners receive a detailed explanation of the study either via the MPB web site (https://www.marspetcarebiobank.com) or from in-hospital information brochures. Answers to frequently asked questions are provided and participants have access to a helpdesk to support any queries. Owners expressing interest online are directed to their veterinary hospital where appointments are scheduled. At the appointment there is the opportunity to ask questions of the veterinary staff before consent is given. Due to the rapid development of technology and long-term nature of the study, renewed consent will be sought over time, as uses of data, samples or participant's circumstances change. To benefit the participating pets, blood biochemistry and haematology results are returned to the attending veterinarian and Wisdom Panel™ (Wisdom Health, Vancouver, WA, USA) genotyping results directly to the pet owner. In alignment with many human biobanks [32], all other results e.g., whole genome sequences, are not made generally available to the veterinary team or to the owner to avoid misinterpretation of non-clinically validated data. During consent, participants are clearly informed about which individual results are returned and are advised of their right to withdraw at any time without explanation, penalty, or influence on their pet’s veterinary care.

### Eligibility

The aim of the MPB is to enrol healthy pets and to follow them throughout their lives, where some will remain healthy, and others will go on to develop illnesses and chronic conditions. All existing healthy patients of MPB study sites, between the ages of 6 months and 10 years for dogs, or 12 years for cats, are eligible. Male and female, neutered or entire cats and dogs with a body weight ≥ 2.5 kg (to ensure the required blood volume is well within 5% of circulating volume) may be included if their body condition score (BCS) is 3—7 on a 9 point scale [33, 34]. All recruits must be in apparent good health without diagnosed or strongly suspected acute or chronic medical conditions. Inclusion and exclusion criteria are provided in Additional file 1. These criteria guide the recruitment of healthy pets, whilst it is accepted that those with sub-clinical conditions may be deemed ‘healthy’ this is informative to the study. Blood biochemistry or haematology results leading to a strong clinical suspicion of underlying disease after the enrollment visit are considered a screening failure. Enrolled pets that subsequently develop health conditions continue to participate at the discretion of the owner and veterinarian assuming that there is no medical or behavioural condition that prevents their continued involvement. For example, conditions such as severe anemia, haemostatic disorders, severe skin disease and stress-related aggression, could preclude continued participation due to unsuitability for blood sampling.

### Data collection

**Fig. 2:**
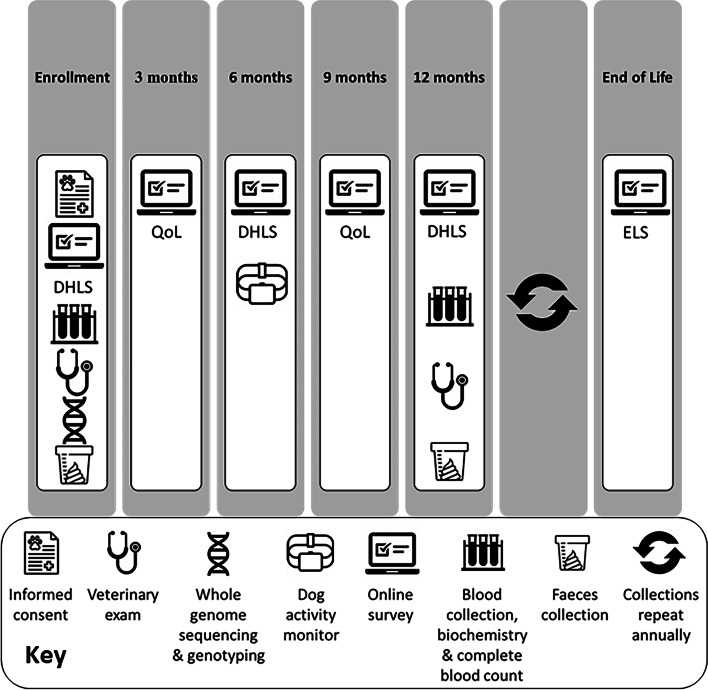
Schematic timeline of the MARS PETCARE BIOBANK data collections. DHLS: diet health and lifestyle survey, QoL; quality of life, ELS: end of life survey

Data types collected are summarised in Table 1 and Fig. 2. Alongside the EMR, the primary instrument used to collect participant information is the DHL survey administered via the EDC system; a list of DHL question categories is shown in Table 2. After enrollment, owners are asked to provide baseline information on their pet’s behaviour, diet, physical activity, lifestyle, and environment as well as completing a separate QoL survey [35]. This is completed every 3 months and the DHL surveys 6-monthly to identify changes in diet, environment, behaviour, activity, medication, and overall health status between annual veterinary visits (Fig. 2). Reminder emails are sent to owners seven and fourteen days after the survey is sent to them. At the time of writing, the completion rates for the enrollment surveys for dog and cat owners are 58.0% and 57.5% respectively. After six months of participation, dog owners are offered a collar-worn accelerometer (Whistle™, San Francisco, CA, USA) to be worn for as long as the owner wishes, with consent to allow the MPB access to the resulting data. This timing is to allow for withdrawal of any pets with abnormal blood results after the enrolment visit and to act as an incentive to owners at the time of the six-month survey. An end-of-life survey is also sent to owners if an enrolled pet dies. The study EDC system allows surveys to be easily accessed by owners and for new surveys to be developed to answer research questions.

**Table 1:** MARS PETCARE BIOBANK data sources and biological samples collected

Data source	Description
Owner-reported surveys	
Enrolment DHL	Information about each pet’s behaviour, diet, physical activity, lifestyle, environment and QoL
Quarterly QoL	Canine wellbeing instrument [35]. Feline instrument currently in development
Six-monthly DHL	As at enrolment, plus health, use of medications, supplements and QoL
End-of-Life	Owner-reported cause of death and reasons for euthanasia (if applicable)
Veterinary data	
Study EDC	Veterinarian-recorded data at time of visit; age, sex, breed, weight, BCS, neuter status, supplements, and annual physical exam
Veterinary EMR	Body weight history, date of spay/neutering (if applicable), frequency and reason for veterinary visits, vaccinations, preventive care, medications, test results and procedure history Cause of death or euthanasia if applicable
Biological data	
Annual blood biochemistry and haematology	“Superchem” analysis and complete blood count (catalogue #SA020, Antech Diagnostics, Fountain Valley, CA, USA)
Faecal microbiome	Shallow Shotgun metagenomics Illumina 2 × 150 bp, ~ 5 M reads
Whole genome sequencing	Illumina X30 depth
Genotyping	Wisdom Panel (Wisdom Health, Vancouver, WA, USA)
Activity monitors	Tri-axis canine collar worn accelerometers (Whistle, San Francisco, USA). Received 6 months after enrolment
Biological Samples Banked Annually	
EDTA plasma	Blood collected in Lavender top EDTA tubes (catalogue # 454,024, Greiner Bio-One, Monroe, NC, USA). Plasma prepared by centrifugation at 3,500 RPM for 10 min
Blood cell pellet	Blood cell pellets reserved after EDTA anticoagulated plasma removal
Serum	Blood collected and allowed to clot for 20–30 min in serum separator clot activator tube (catalogue # 454,067, Greiner Bio-One, Monroe, NC, USA). Centrifuged at 3,500 rpm for 10 min
Whole Blood	Whole blood stabilised for RNA in RNAprotect Animal blood tubes (catalogue #76,554; Qiagen, Germantown, MD, USA)
Faeces	Faeces stabilised for DNA using PERFORMAbiome·GUT collection devices (PB-200 DNA Genotek, Inc. Ottawa, Canada)

*DHLS* Diet, Health and Lifestyle Survey, *QoL* Quality of Life, *EDC* Electronic data capture, *EMR* Electronic Medical record, *bp* Base pair, *EDTA* Ethylenediaminetetraacetic acid

**Table 2:** Categories of survey questions included in the MARS PETCARE BIOBANK surveys

Species	Category	Description
Enrolment DHL survey		
Dog & Cat	Pet information	Name, age, sex, neuter status/date, age at, and method of adoption
Dog & Cat	Feeding Habits	Quantity, format and brand of food/s most often fed over previous 2 weeks Feeding pattern Use of treats and type provided
Dog & Cat	Behaviour & Lifestyle	Day time location Night time location Other pets in household Children in household Travel history Exercise habits
Cat	Behaviour & Lifestyle	Outdoor access Hunting Use of cat litter
6 Monthly DHL survey		
Dog & Cat	Pet information	Name, age, sex, neuter status/date
Dog & Cat	Feeding habits	Quantity, format and brand of food fed most often over previous 2 weeks Feeding pattern Use of treats and type provided Change of diet in last 6 months and reason
Dog & Cat	Behaviour & Lifestyle	Day time location Night time location Other pets in household Children in household Travel in last 6 months Exercise habits
Cat specific	Behaviour & Lifestyle	Outdoor access Use of cat litter Hunting
Dog & Cat	Health / clinical signs	Veterinary visits and reason Health signs (e.g., itching, vomiting) OTC medications used OTC supplements used Prescribed medications Owners’ assessment of body condition
End of life survey		
Dog & Cat	Pet information	Name, age,
	End of life	Date of death Cause of death Reason for euthanasia if applicable

*DHL* Diet, Health and Lifestyle, *OTC* Over the counter

Enrolled pets attend their veterinary hospital annually (± 4 months of the anniversary of the enrolment visit) where an examination is carried out and clinical information recorded. Biological samples are collected using standardised protocols and collection tubes for plasma and serum separation, whole blood stabilised for RNA and faeces stabilised for DNA extraction (Table 1). At enrolment, a cheek swab is also collected and sent directly for genotyping according to manufacturer protocols (Wisdom Health, Vancouver, WA, USA) [36, 37]. All other biological samples are transported at 4–8 °C to local Antech Diagnostics laboratories, where routine serum biochemistry and haematology are performed, plasma, serum, and fecal material prepared, aliquoted and all biological samples frozen at -80 °C within 24–36 h of collection. Serum biochemistry includes pancreatic lipase, total protein, albumin, globulin, albumin/globulin ratio, total bilirubin, Gamma-glutamyl transferase, alkaline phosphatase, alanine aminotransferase, aspartate aminotransferase, creatine phosphokinase, phosphorus (P), blood urea nitrogen (BUN), creatinine, BUN/creatinine ratio, glucose, calcium (Ca), magnesium (Mg), sodium (Na), potassium (K), Na/K ratio, chloride (Cl), cholesterol, triglycerides, amylase and symmetric dimethylarginine (SDMA). The complete blood count measures white blood cell count, red blood cell count, haemoglobin, haematocrit, mean corpuscular volume, mean corpuscular haemoglobin concentration, mean corpuscular haemoglobin, and platelet count with a complete differential. If sample collection is not successful a follow up visit is scheduled within 3 weeks. Frozen biological samples are shipped in batches at -80 °C to the MPB facility at Antech Diagnostics, Brownsburg, IN, USA, for long-term storage. For each participating pet, DNA is extracted from enrollment blood cell pellets for whole genome sequencing (WGS, Illumina X30 depth, Table 1). DNA is also extracted from banked stabilised faecal material for Shallow Shotgun Metagenomic analysis (Illumina 2 × 150 bp, ~ 5 M reads, Table 1). Protocols for biological sample collection, transport and handling were previously piloted to determine quality requirements. Pre/post-analytical quality data including collection and freezing time, transportation conditions, and analytical quality control are recorded in an integrated Laboratory Information Management System proprietary to Antech Diagnostics.

To enable analysis, all data are collected into a central data repository, based on the Microsoft Azure solid hyperscale cloud platform and its Azure Data Lake Store. For long term retention, data is ingested in its raw format via the service providers’ Application Programming Interface, direct database connections or Secure File Transfer Protocols. The data repository provides a secure and flexible workspace for data transformation and analytics via scalable compute engines offering clear lineage and discovery for all datasets produced. Stored data is encrypted and leverages the Azure Active Directory security model for access to any data in its folder structures. In compliance with data protection regulations including those applicable in the United States, United Kingdom, and the European Union, all personally identifiable information is removed and stored in an encrypted location.

### Statistical analysis

Univariate or multivariable modelling techniques will be used to identify and quantify the effects of novel genetic and environmental risk factors for a range of phenotypes. To avoid the possible issue of identifying small non-clinically relevant but statistically significant effects, the biologically relevant effect size will be defined prior to testing a hypothesis. When identifying an effect from an exploratory perspective, the biological relevance will be established using other sources. Making use of the repeated measurements available both before and after diagnoses, predictive modelling such as that detailed in Bradley et al. 2019 [38] will be used for the early detection of medical conditions.

### Power calculations

The incidence of specific health conditions or phenotypes in the MPB population will be determined at regular intervals (approximately every 3 years) and power calculations will vary according to the phenotype of interest, the age profile of recruited pets, the estimated sample size and prevalence within the dataset. To inform recruitment and retention targets, modelling was performed of the time taken to recruit phenotypes of interest, e.g., overweight and obesity, based on the cat and dog populations attending Banfield Pet Hospitals in 2021. A data frame for the eligible Banfield Hospital population was obtained using the MPB inclusion criteria and a maximum age of 20 years. This contained 1,155,818 cats, 274,972 with a diagnosis of obesity or overweight and 5,825,713 dogs of which 1,301,985 had a diagnosis of obesity or overweight. The cumulative hazard function for the likelihood of a diagnosis of overweight or obesity was calculated by the Nelson-Aalen estimator [39] (Additional file 2) and used to randomly simulate datasets for 500 biobank populations with 10 years of recruitment. A range of recruitment and retention rates informed by the experience of the VCA Animal Hospitals Clinical Studies team and available literature [21, 40, 41] were applied. These included, four fixed loss to follow up (LTFU) rates of 10, 20, 30 or 40% every year (Additional files 3 and 4) and three tiered LTFU scenarios (Additional files 5 and 6). A conservative LTFU scenario of 10% after year 1, 5% annually in years 3 to 5, then 10% annually in years 6 to 8 followed a 20% annual loss in years 9 and 10 due to increased mortality as pets age. A moderate LTFU scenario of 15% after year 1, 5% annually in years 3 and 4, followed by 10% annually in years 5 and 6 and a 20% annual loss from year 7 onwards due to increased mortality as pets age. Finally, a high LTFU scenario of 20% after year 1, 10% annually in years 3 and 4, followed by 15% in years 5 and 6 then a 20% annual loss from year 7 onwards due to increased mortality as pet age. The mean (95% confidence interval) number of pets in the population with this phenotype after each year of recruitment was calculated. Accordingly, we estimate that it will take at least 4 -5 years to have sufficient numbers of dogs and cats within the cohort to allow the investigation of possible risk factors of obesity or overweight (Additional files 3, 4, 5 and 6). This assumes, based on available research [42–44], the requirement for a minimum of 100 cases with three data and biological sample collections prior and one collection post diagnosis. As the study population increases and long-term follow up is carried out, the study of less prevalent but still common conditions will be possible (e.g., canine osteoarthritis, feline hyperthyroidism).

### Project oversight

The study coordination team, consisting of representatives of the contributing Mars Petcare business units, ensures that the operational details of the MPB are performed appropriately. During the development process and as required, this team is supported by working groups established to address specific operational areas such as sample processing and storage. The study objectives and budgets are monitored and approved by the steering team, including the Chief Medical, Data and Financial Officers, and the Vice Presidents of Science, and Research and Development for Mars Petcare. The study design, execution, and ethical review (owner informed consent and standard of care) were approved by the MVH Internal Review Board (IRB) a designated review board independent of any other authority within the organisation and given ultimate authority to approve, require modifications to, or reject research proposals. The IRB follows the guidelines of the American Veterinary Medical Association, UK Royal College of Veterinary Surgeons, and the Clinical and Translational Science Award One Health Alliance (COHA) for the review of studies involving client-owned animals. The IRB comprises, independent members including Diplomates of the American College of Laboratory Animal Medicine, a veterinary bioethicist, lay people and scientific and veterinary subject matter experts from Mars Petcare.

## Discussion

Researchers within Mars Petcare are experienced in generating scientific insights from large-scale datasets extracted from EMRs [45–47]. For example, advanced machine learning methods combined with large sets of health screening data were recently used to develop models to predict early risk of chronic kidney disease in cats and dogs [38, 48]. The systematic collection of a wider range of data and biological samples will provide a much deeper understanding of companion animal health. To this end, the MPB has been designed to develop a longitudinal dataset of DHL information combined with concurrent annual biological sample sets to facilitate association studies between conditions and a wide variety of potential risk factors. It is expected that these data will yield predictive and prognostic biomarkers of disease and important insights into companion animal health over the coming years. Areas for investigation will include:

- The development and progression of renal, neurologic, and cardiac disorders in companion animal ageing.
- The incidence and risk factors for common conditions including obesity, infectious disease, orthopaedic disorders, and cancers.
- The influence of diet and the gastrointestinal microbiome on health and disease.
- The identification of candidate loci for genetic variants associated with diseases and their interactions with lifestyle and environmental factors through genome wide association studies.

To support increased understanding of companion animal genomics the MPB will release raw WGS data and variant calling files to the publicly available NCBI Archive with the required attributes. It is hoped that research using such data will accelerate the emerging field of personalised veterinary medicine, aiding the development of screening and confirmatory diagnostic tests to assist in treatment decisions and identify targeted therapies. This will be particularly impactful in feline medicine, which is under supported by longitudinal cohort studies. Although the MPB is observational in nature, in the future, there may be opportunities for nested or parallel interventional studies to investigate specific hypotheses.

A strength of the MPB is its ability to leverage the Mars Petcare network of veterinary healthcare, diagnostics and direct to consumer service providers to overcome many of the challenges encountered in establishing such a project. This shared oversight also allows increased harmonisation of data types to enhance interoperability and add further value. The large target sample size of the project is also a major strength, and we aim to achieve this and promote retention by applying a range of engagement initiatives, including the development of a MPC Biobank community for participants to engage with each other and to allow the study to share research outcomes. Enrolment from the general dog and cat population rather than from particular breeds will enable subsequent research to deliver insights applicable to the widest number of pets, although the application of genetic analyses may be complicated by the presence of mixed as well as pure breeds. Furthermore, by recruiting a broad age range of healthy pets, we hope to shorten the number of years required to begin accruing disease phenotypes of interest, while still addressing questions around healthy ageing and the influence of lifestyle and diet. This strategy does, however, entail a trade off in the number of years of data available per pet and losses due to mortality particularly in large breeds dog which may have a shorter lifespan than small breed dogs. The collection of owner-reported data enables diet, health and lifestyle risk factors not necessarily recorded in EMR to be investigated. Furthermore, the use of a validated, robust QoL survey tool [35] will provide key insights into aspects of animal health and welfare that are not typically collected, including proxy assessment of quality of life.

The limitations of the study are that despite the planned size of the cohort, there may not be sufficient statistical power to investigate low prevalence phenotypes. Even in a planned cohort of 10,000 individuals per species, it will be several years before enough animals are recruited to allow reliable statistical analyses for any particular disease. Recruitment may be affected by negative perception of industry involvement in biobanking; reported to be a significant risk to human initiatives [49, 50]. Research suggests that prejudice against industry involvement can be alleviated by demonstrating clear oversight and governance processes and that subsequent research will be driven by the goal of mutual benefit [49, 50], as exemplified by the MPB. Inherent in all large-scale, multicentre studies, is some variability in examination and diagnoses. To minimise this, study specific training is given to all veterinary professionals taking part and a detailed protocol and in-hospital EDC are in place to ensure the same data is recorded in the same way at each visit. As recruitment is through primary veterinary practices where a full clinical work up to confirm exclusionary conditions is not possible or may not be supported by the owner, some degree of subjectivity is likely to occur. To address this, when data analysis takes place, factors such as operator and visit site will be considered to assess the effect of intra and inter site variability. Owner-reported data, particularly relating to health, may be unreliable or biased, although as EMR are also available the degree of information bias can be assessed. Bias resulting from differential selection of owners, in terms of location, and socio-economic status (determined from hospital location) and of pets, in terms of age, gender, breed etc. is a common factor in all biobank initiatives and could occur here. However, the population demographics will be evaluated statistically at regular intervals and addressed through targeted recruitment strategies if appropriate. Also, some bias may be inherent in recruiting through veterinary hospitals as only those owners willing and able to engage with regular veterinary care are likely to enrol and maintain participation. If an enroled pet changes location, they may continue the study attending a participating hospital in their new area, although if this is not possible, they will unfortunately be lost to follow up. While every effort is made to recruit only healthy pets, there is the possibility of recruiting those with subclinical conditions but as such they will be informative to the aim of investigating early biomarkers of disease. Furthermore, the exclusion, on welfare grounds, of enrolled pets that develop health or behavioural conditions that prevent their continued involvement, could preclude investigation of the progression, treatment, and outcome of such diseases and conditions. The biological sample types chosen for collection and storage aim to provide maximum scientific return within the principles of the 3R’s (Replacement, Reduction and Refinement) [51] and the available budget. Analyses not currently included as part of the protocol such as tests for heartworm or feline leukemia virus (FeLV) and feline immunodeficiency virus (FIV) could be carried out retrospectively using stored material. Processing and storage methods that may restrict sample use in current or future applications have been avoided as far as possible. However, not all biological sample types or possible applications will be covered by the protocol. Collection of other biological samples (hair, nails, saliva, and urine) were considered, but excluded at launch either due to their limited utility compared to blood, or difficulty of collection. The addition of such biological sample types may be possible in the future if required for specific research applications. Over a study of this length some analysis methodologies are likely to change, and cross method validation will be put in place to account for this where possible. For genomic data, reanalysis of existing data using new bioinformatics methods will be carried out where applicable. Careful record keeping and quality management processes are in place to record method versions and changes will be included as covariates in any analyses. Though typical of most biobanks, a perceived limitation of the MPB may be that not all data and biological samples will be immediately available via unrestricted open access. However, researchers wishing to initiate research based on the MPB are invited to contact the Biobank (info@marspetcarebiobank.com) with proposals. Mars Petcare encourages and has a long history of publishing research findings in peer reviewed journals. To support this ambition, anonymised data sets will be made available concomitant with publication. In conclusion, by providing extensive new data to elucidate the risk factors and processes that determine the development of disease in cats and dogs, the MPB will provide a unique opportunity to advance precision and personalised veterinary healthcare.

### Supplementary Information

**Additional file 1: Supplementary Table S1.** Inclusion and exclusion criteria.

**Additional file 2. **Cumulative hazard for a diagnosis of obesity or overweight for cats and dogs.

**Additional file 3.**

**Additional file 4.**

**Additional file 5.**

**Additional file 6.**

## Data Availability

All data generated or analysed during the development of this study are included in this published article and its supplementary information files. De-identified, raw whole genome sequence data will be made available in a suitable public archive such as the NCBI Sequence Read Archive. De-identified variant calling data (VCF) are subject to a 12-month embargo from the date of creation, after this time the VCF will also be available in a suitable public archive. Researchers wishing to initiate research based on the MPB are invited to contact the Biobank (info@marspetcarebiobank.com) to obtain access for specific research proposals.

## Abbreviations

MPB: MARS PETCARE BIOBANK
ISBER: International Society for Biological and Environmental Repositories
EMR: Electronic medical record
MVH: Mars Veterinary Health
QoL: Quality of life
DHL: Diet, health, and lifestyle
DHLS: Diet, health, and lifestyle survey
EDC: Electronic data capture system
BCS: Body condition score
BUN: Blood urea nitrogen
SDMA: Symmetric dimethylarginine
WGS: Whole genome sequencing
LTFU: Lost to follow up
IRB: MVH Internal Review Board
COHA: Clinical and Translational Science Award One Health Alliance
3R’s: Replacement, Reduction and Refinement
ELS: End of life survey
OTC: Over the counter
bp: Base pair
EDTA: Ethylenediaminetetraacetic acid
VCF: Variant calling files
