# Supplementary material for: The MARS PETCARE BIOBANK protocol: establishing a longitudinal study of health and disease in dogs and cats
Source: BMC Vet Res. 2023 Aug 17;19:125. doi: 10.1186/s12917-023-03691-4 (PMC10433631; doi:10.1186/s12917-023-03691-4)
Supplement: Supplementary file 1 — Additional file 1: Supplementary Table S1. Inclusion and exclusion criteria. [file 12917_2023_3691_MOESM1_ESM.docx]

Supplementary Material

Supplementary table S1: Inclusion and exclusion criteria

| Dog | |
| --- | --- |
| Inclusion criteria | Exclusion criteria at enrollment |
| - Existing client of an MPB^a^ study site - Age: 6 months -10 years - Sex: male or female - Neutered or entire/intact - Body weight: > 2.53 kg - BCS^b^: 3-7 of 9 - In apparent good health without any clinically obvious or strongly suspected uncontrolled acute or chronic medical conditions. - Masses or tumors known to be benign (by fine needle aspirate and cytology or biopsy with histopathology) and unknown neoplasia not strongly suspected to be malignant based on clinical behaviour or location. - Osteoarthritis managed only with supplements. - Mild to moderate dermatopathies not being treated with prescription medication. - Dental disease with mild to moderate tartar and/or up to stage 2 periodontal disease and previous dental extractions (unless associated with oral cancer) permitted). - Behavioral conditions not being managed with prescription medications. - Supplements and OTC^d^ medications permitted. | - Previous diagnosis or treatment of concurrent disease, e.g., hypothyroidism, hyperthyroidism, kidney disease, hepatopathy, chronic pancreatitis, diabetes, hyperadrenocorticism, hypoadrenocorticism, “Metabolic Disorder”, exocrine pancreatic insufficiency, infectious disease, autoimmune disease, cardiac disease, and malignant neoplasia. - A presumptive diagnosis of a medical condition based on clinical exam or history that would present an exclusion including heart murmurs unless known to not result in hemodynamic consequences. - In receipt of prescription medication, with the exception of regular prophylactic flea, tick, and deworming (including heartworm) treatments. - Minor ailments including infectious diseases and parasites under curative therapy (e.g., infectious upper respiratory tract disease, kennel cough, worms, etc.), as well as those receiving medication for minor injuries (e.g., NSAIDs^c^, antibiotics), while treatment is ongoing until a minimum of 60 days after completion of treatment, and assuming the inclusion criteria are satisfied. - Pregnancy and lactation until a minimum of 12 weeks post-partum. |
| Cat | |
| Inclusion criteria | Exclusion criteria at enrollment |
| - Existing client of an MPB study site - Age 6 months -12 years - Sex: male or female - Neutered or entire/intact - Body weight: > 2.53 kg - BCS: 3-7 of 9 - In apparent good health without any clinically obvious or strongly suspected uncontrolled acute or chronic medical conditions. - Supplements and OTC medications are permitted. - Dental disease with mild to moderate tartar and/or up to stage 2 periodontal disease and previous dental extractions (unless associated with oral cancer or feline gingivostomatitis, i.e., full mouth) permitted. - Osteoarthritis managed only with supplements. - Masses or tumors known to be benign (by fine needle aspirate and cytology or biopsy with histopathology) and unknown neoplasia not strongly suspected to be malignant based on clinical behaviour or location. - Mild to moderate dermatopathies not being treated with prescription medication. - Behavioral conditions not being managed with prescription medications. - Positive or unknown retroviral status in cats unless concurrent clinical disease is present or strongly suspected. | - Previous diagnosis or treatment of concurrent disease, for example: hypothyroidism, hyperthyroidism, kidney disease, hepatopathy, chronic pancreatitis, diabetes, hyperadrenocorticism, hypoadrenocorticism, “Metabolic Disorder”, exocrine pancreatic insufficiency, infectious disease, autoimmune disease, cardiac disease, and malignant neoplasia. - A presumptive diagnosis of a medical condition based on clinical exam or history that would present an exclusion including heart murmurs unless known to not result in hemodynamic consequences. - Palpable goiter or markedly thickened intestines even if no diagnosis has been made at the time of the clinical exam. - In receipt of prescription medication, with the exception of regular prophylactic flea, tick, and deworming (including heartworm) treatments. - Minor ailments including infectious diseases and parasites under curative therapy (e.g., infectious upper respiratory tract disease, worms, etc.), as well as those receiving medication for minor injuries (e.g., NSAIDs, antibiotics), are excluded while treatment is ongoing util a minimum of 60 days after completion of treatment, and assuming the inclusion criteria are satisfied. - Known Heartworm positive. - Pregnancy and lactation until a minimum of 12 weeks post-partum. |
| Exclusion criteria for both species following enrollment | |
|  | - At the discretion of the attending veterinarian or in the event of a new medical or behavioural condition that prevents safe blood collection e.g., a bleeding disorder or aggression. - Results of blood biochemistry or haematology undertaken at the enrollment visit leading to a strong clinical suspicion of an underlying illness or disease, is considered a screening failure. - Pregnancy: no blood samples collected until at least 12 weeks post-partum. - Participants that permanently move to a hospital that is not an MPB recruiting site. |

a; MPB: MARS PETCARE BIOBANK, b; BCS: body condition score, c; NSAIDS: non-steroidal anti-inflammatory drugs, d; OTC: Over the counter
