# Supplementary figures and images for: The MARS PETCARE BIOBANK protocol: establishing a longitudinal study of health and disease in dogs and cats
Source: BMC Vet Res. 2023 Aug 17;19:125. doi: 10.1186/s12917-023-03691-4 (PMC10433631; doi:10.1186/s12917-023-03691-4)

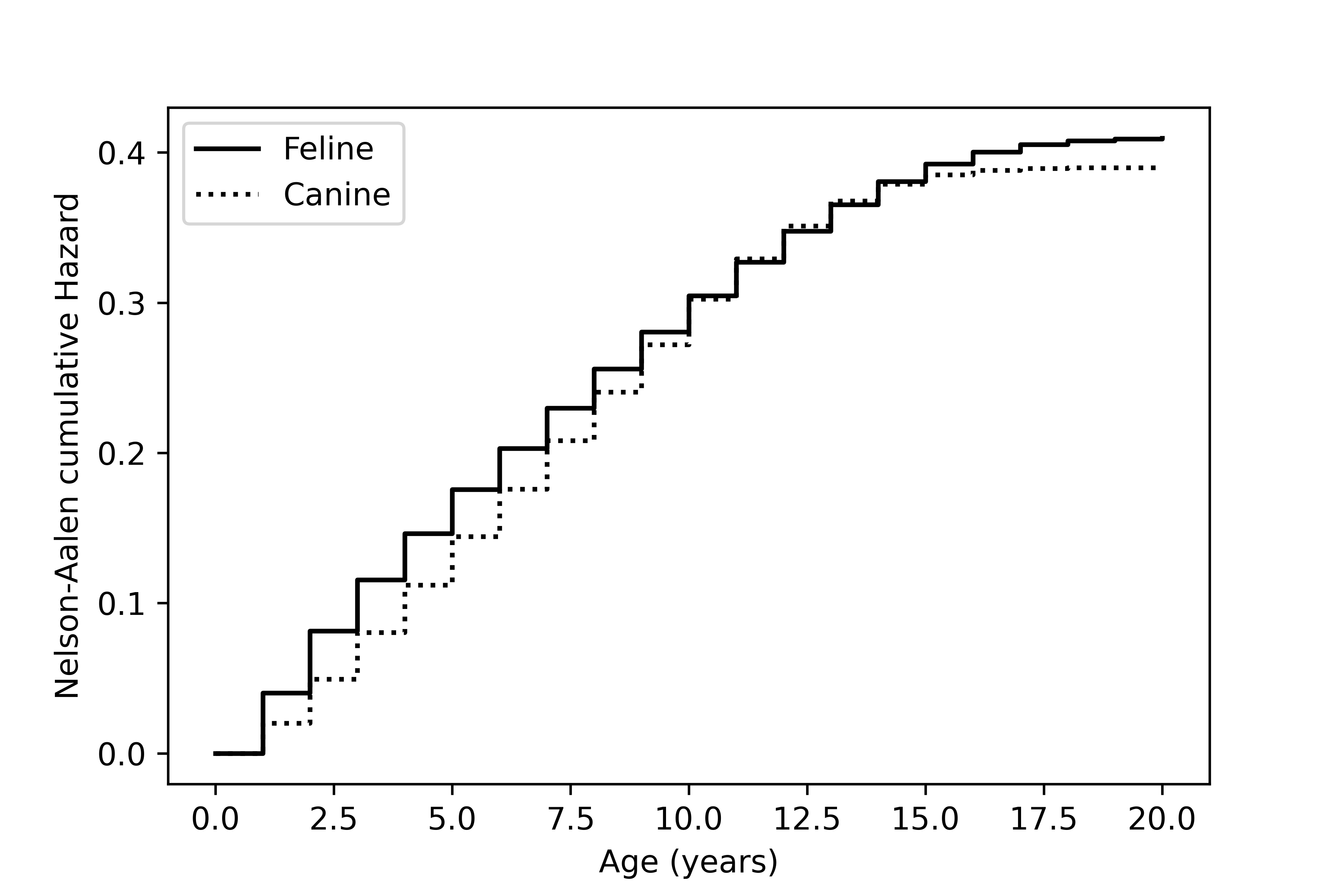

Supplement: Supplementary file 2 — Additional file 2. Cumulative hazard for a diagnosis of obesity or overweight for cats and dogs. [file 12917_2023_3691_MOESM2_ESM.tiff]
