# Supplementary material for: The MARS PETCARE BIOBANK protocol: establishing a longitudinal study of health and disease in dogs and cats
Source: BMC Vet Res. 2023 Aug 17;19:125. doi: 10.1186/s12917-023-03691-4 (PMC10433631; doi:10.1186/s12917-023-03691-4)
Supplement: Supplementary file 3 — Additional file 3. [file 12917_2023_3691_MOESM3_ESM.docx]

Supplementary Table S2.

| Recruitment year | Mean (95% CI) number of dogs with 3 pre and 1 post diagnosis sample^a^ | | | |  |
| --- | --- | --- | --- | --- | --- |
|  | 10 % LTFU^b^ | 20% LTFU | 30% LTFU | 40% LTFU | |
| 1 | 0.0 | 0.0 | 0.0 | 0.0 | |
| 2 | 0.0 | 0.0 | 0.0 | 0.0 | |
| 3 | 0.0 | 0.0 | 0.0 | 0.0 | |
| 4 | 100.2 (99.7, 100.6) | 63.0 (62.6, 63.5) | 34.0 (33.5, 34.4) | 15.5 (15.1, 15.8) | |
| 5 | 344.6 (343.8, 345.4) | 215.2 (214.3, 216.1) | 115.6 (114.7, 116.6) | 49.8 (49.1, 50.5) | |
| 6 | 706.1 (705.0, 707.2) | 434.1 (432.8, 435.4) | 226.6 (225.2, 228.1) | 93.8 (92.8, 94.8) | |
| 7 | 1154.2 (1152.7, 1155.7) | 699.5 (697.8, 701.2) | 352.9 (351.0, 354.8) | 141.1 (139.8, 142.5) | |
| 8 | 1664.1 (1662.3, 1666.0) | 991.2 (989.1, 993.4) | 486.0 (483.7, 488.2) | 189.5 (187.9, 191.0) | |
| 9 | 2216.9 (2214.6, 2219.1) | 1297.0 (1294.5, 1299.5) | 622.0 (619.4, 624.5) | 238.6 (236.9, 240.4) | |
| 10 | 2793.7 (2791.1, 2796,3) | 1607.9 (1607.1, 1612.9) | 758.5 (755.7, 761.3) | 287.0 (285.1, 288.9) | |

The mean number of dogs (95% confidence interval) in the population with a diagnosis of obesity or overweight after each year of recruitment calculated from 500 simulated populations using four fixed percentage loss to follow up rates. ^a^ Based on targeted recruitment rates of 1000 healthy dog per year. ^b^ LTFU; Loss to follow up.
