# Supplementary material for: The MARS PETCARE BIOBANK protocol: establishing a longitudinal study of health and disease in dogs and cats
Source: BMC Vet Res. 2023 Aug 17;19:125. doi: 10.1186/s12917-023-03691-4 (PMC10433631; doi:10.1186/s12917-023-03691-4)
Supplement: Supplementary file 4 — Additional file 4. [file 12917_2023_3691_MOESM4_ESM.docx]

Supplementary Table S3.

| Recruitment year | Mean (95% CI) number of cats with 3 pre and 1 post diagnosis sample^a^ | | | |  |
| --- | --- | --- | --- | --- | --- |
|  | 10 % LTFU^b^ | 20% LTFU | 30% LTFU | 40% LTFU | |
| 1 | 0.0 | 0.0 | 0.0 | 0.0 | |
| 2 | 0.0 | 0.0 | 0.0 | 0.0 | |
| 3 | 0.0 | 0.0 | 0.0 | 0.0 | |
| 4 | 62.9 (62.4, 63.3) | 33.7 (33.3, 34.1) | 15.2 (14.9, 15.5) | 5.8 (5.6, 6.1) | |
| 5 | 256.1 (255.3, 256.9) | 135.3 (134.4, 136.2) | 59.5 (58.8, 60.2) | 20.7 (20.2, 21.2) | |
| 6 | 567.6 (566.4, 568.7) | 289.7 (288.3, 291.1) | 122.9 (121.7, 124.0) | 40.3 (39.6, 41.1) | |
| 7 | 965.4 (963.7, 967.0) | 477.6 (475.7, 479.5) | 195.6 (194.1, 197.1) | 61.1 (60.1, 62.0) | |
| 8 | 1421.2 (1419.1, 1423.3) | 686.0 (683.6, 688.4) | 272.3 (270.5, 274.2) | 82.1 (81.0, 83.2) | |
| 9 | 1910 (1907.6, 1912.7) | 905.4 (902.6, 908.2) | 350.4 (348.2, 352.5) | 103.0 (101.8, 104.3) | |
| 10 | 2418.7 (2415.7, 2421.6) | 1129.4 (1126.3, 1132.6) | 428.9 (426.6, 431.2) | 124.0 (122.7, 125.4) | |

The mean number of cats (95% confidence interval) in the population with a diagnosis of obesity or overweight after each year of recruitment calculated from 500 simulated populations using four fixed percentage loss to follow up rates. ^a^ Based on targeted recruitment rates of 1000 healthy cats per year. ^b^ LTFU; Loss to follow up.
