# Supplementary material for: The MARS PETCARE BIOBANK protocol: establishing a longitudinal study of health and disease in dogs and cats
Source: BMC Vet Res. 2023 Aug 17;19:125. doi: 10.1186/s12917-023-03691-4 (PMC10433631; doi:10.1186/s12917-023-03691-4)
Supplement: Supplementary file 5 — Additional file 5. [file 12917_2023_3691_MOESM5_ESM.docx]

Supplementary Table S4.

| Recruitment year | Mean (95% CI) number of dogs with 3 pre and 1 post diagnosis sample ^a^ | | |
| --- | --- | --- | --- |
|  | Conservative LTFU^b^ | Moderate LTFU^c^ | High LTFU^d^ |
| 1 | 0.0 | 0.0 | 0.0 |
| 2 | 0.0 | 0.0 | 0.0 |
| 3 | 0.0 | 0.0 | 0.0 |
| 4 | 113.1 (112.7, 113.5) | 100.0 (99.5, 100.4) | 83.8 (83.3, 84.2) |
| 5 | 335.1 (334.4, 335.9) | 282.3 (281.5, 283.2) | 199.2 (198.2, 200.1) |
| 6 | 600.2 (599.1, 601.4) | 474.1 (472.9, 475.4) | 271.8 (270.5, 273.1) |
| 7 | 839.3 (837.8, 840.9) | 611.4 (609.8, 613.0) | 291.6 (290.1, 293.0) |
| 8 | 991.8 (989.9, 993.8) | 659.8 (658.1, 661.6) | 292.1 (290.7, 293.6) |
| 9 | 1057.0 (1054.8, 1059.2) | 663.4 (661.7, 665.2) | 292.1 (290.7, 293.6) |
| 10 | 1068 (1066.4, 1071.0) | 663.5 (661.7, 665.2) | 292.1 (290.7, 293.6) |

# The mean number of dogs (95% confidence interval) in the population with a diagnosis of obesity or overweight after each year of recruitment calculated from 500 simulated populations and three differing models of loss to follow up (LTFU).

^a^ Based on targeted recruitment rates of 1000 healthy dogs per species per year

^b^ Conservative LTFU of 10% after year 1, a reduced loss thereafter of 5% annually in years 3 to 5, then 10% annually in years 6 to 8 followed by a 20% annual loss in years 9 and 10 due to increased mortality as dogs age.

^c^ Moderate LTFU of 15% after year 1, a reduced loss thereafter of 5% annually in years 3 and 4, followed by 10% annually in years 5 and 6 and a 20% annual loss from year 7 onwards due to increased mortality as dogs age.

^d^ High levels of LTFU; 20% after year 1, a reduced loss thereafter of 10% annually in years 3 and 4, followed by 15% in years 5 and 6 then a 20% annual loss from year 7 onwards due to increased mortality as dogs age.
