# Supplementary material for: The MARS PETCARE BIOBANK protocol: establishing a longitudinal study of health and disease in dogs and cats
Source: BMC Vet Res. 2023 Aug 17;19:125. doi: 10.1186/s12917-023-03691-4 (PMC10433631; doi:10.1186/s12917-023-03691-4)
Supplement: Supplementary file 6 — Additional file 6. [file 12917_2023_3691_MOESM6_ESM.docx]

Supplementary Table S5.

| Recruitment year | Mean (95% CI) number of cats with 3 pre and 1 post diagnosis sample ^a^ | | |
| --- | --- | --- | --- |
|  | Conservative LTFU^b^ | Moderate LTFU^c^ | High LTFU^d^ |
| 1 | 0.0 | 0.0 | 0.0 |
| 2 | 0.0 | 0.0 | 0.0 |
| 3 | 0.0 | 0.0 | 0.0 |
| 4 | 76.8 (76.4, 77.2) | 66.1 (65.6, 66.6) | 52.9 (52.4, 53.4) |
| 5 | 250.7 (249.9, 251.5) | 199.8 (198.8, 200.7) | 128.7 (127.8, 129.6) |
| 6 | 459.7 (458.4, 461.0) | 334.9 (333.4, 336.3) | 171.1 (169.9, 172.2) |
| 7 | 629.5 (627.7, 631.3) | 423.0 (421.2, 424.8) | 178.9 (177.7, 180.1) |
| 8 | 726.9 (724.8, 729.0) | 448.9 (447.0, 450.7) | 179.1 (177.9, 180.3) |
| 9 | 760.4 (758.2, 762.6) | 450.0 (448.2, 451.9) | 179.1 (177.9, 180.3) |
| 10 | 764.3 (762.1, 766.5) | 450.0 (448.2, 451.9) | 179.1 (177.9, 180.3) |

# The mean number of cats (95% confidence interval) in the population with a diagnosis of obesity or overweight after each year of recruitment calculated from 500 simulated populations and three differing models of loss to follow up (LTFU).

^a^ Based on targeted recruitment rates of 1000 healthy cats per species per year

^b^ Conservative LTFU of 10% after year 1, a reduced loss thereafter of 5% annually in years 3 to 5, then 10% annually in years 6 to 8 followed by a 20% annual loss in years 9 and 10 due to increased mortality as cats age.

^c^ Moderate LTFU of 15% after year 1, a reduced loss thereafter of 5% annually in years 3 and 4, followed by 10% annually in years 5 and 6 and a 20% annual loss from year 7 onwards due to increased mortality as cats age.

^d^ High levels of LTFU; 20% after year 1, a reduced loss thereafter of 10% annually in years 3 and 4, followed by 15% in years 5 and 6 then a 20% annual loss from year 7 onwards due to increased mortality as cats age.
